# Moiré Lattice of Twisted Bilayer Graphene as Template for Non‐Covalent Functionalization

**DOI:** 10.1002/anie.202414593

**Published:** 2024-12-12

**Authors:** Tobias Dierke, Stefan Wolff, Roland Gillen, Jasmin Eisenkolb, Tamara Nagel, Sabine Maier, Milan Kivala, Frank Hauke, Andreas Hirsch, Janina Maultzsch

**Affiliations:** ^1^ Department of Physics Chair of Experimental Physics Friedrich-Alexander-Universität Erlangen-Nürnberg Staudtstr. 7 91058 Erlangen Germany; ^2^ Department of Chemistry and Pharmacy Chair of Organic Chemistry II & Center of Advanced Materials and Processes Friedrich-Alexander-Universität Erlangen-Nürnberg Nikolaus-Fiebiger-Str. 10 91058 Erlangen Germany; ^3^ Organisch-Chemisches Institut Universität Heidelberg Im Neuenheimer Feld 270 69120 Heidelberg Germany

**Keywords:** 2D materials, Density functional calculations, Functionalization, Graphene, Raman spectroscopy

## Abstract

We present a novel approach to achieve spatial variations in the degree of non‐covalent functionalization of twisted bilayer graphene (tBLG). The tBLG with twist angles varying between ~5° and 7° was non‐covalently functionalized with 1,4,5,8,9,11‐hexaazatriphenylenehexacarbonitrile (HATCN) molecules. Our results show a correlation between the degree of functionalization and the twist angle of tBLG. This correlation was determined through Raman spectroscopy, where areas with larger twist angles exhibited a lower HATCN peak intensity compared to areas with smaller twist angles. We suggest that the HATCN adsorption follows the moiré pattern of tBLG by avoiding AA‐stacked areas and attach predominantly to areas with a local AB‐stacking order of tBLG, forming an overall ABA‐stacking configuration. This is supported by density functional theory (DFT) calculations. Our work highlights the role of the moiré lattice in controlling the non‐covalent functionalization of tBLG. Our approach can be generalized for designing nanoscale patterns on two‐dimensional (2D) materials using moiré structures as a template. This could facilitate the fabrication of nanoscale devices with locally controlled varying chemical functionality.

## Graphical abstract

Utilization of the morié lattice formed by twisted bilayer graphene as template for non‐covalent functionalization with HATCN molecules confirmed by Raman spectroscopy measurements and density functional theory calculations.

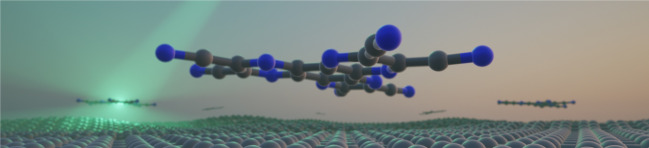

## Introduction

The ability to manipulate graphene by chemical functionalization has opened up a new field of research, including structuring of graphene areas into spatial patterns. A variety of approaches can be used to pattern the basal plane of graphene. For instance, spatial patterning of covalently functionalized graphene can be achieved by the use of plasma jets, both with and without the use of masks,[^1^, ^2^] by generating reactive radicals through laser irradiation,[^3^, ^4^] or by patterning the underlying substrate on which graphene is deposited.^[5]^ However, the challenge lies in controlling the spatial attachment of the functional groups on a nanometer scale,[^4^, ^5^] which is a key requirement for exploiting the full potential of graphene‐based devices. On the other hand, well‐defined nanoscale patterns were successfully achieved by self‐assembly of carbon‐based molecules via non‐covalent interactions on graphene by nanomanipulation in ambient scanning tunneling microscopy (STM).[^6^, ^7^] Such non‐covalent interactions via π ‐π stacking are becoming a focus of recent research.

Twisted bilayer graphene (tBLG) with its moiré superlattice exhibits periodic changes in the local stacking order and electronic band structures, leading to remarkable phenomena such as flat bands,^[8]^ correlated insulating states,[^9^, ^10^] and superconductivity,[^11^, ^12^] thus invoking much research in the field of twisted bilayers, twistronics and moiré structures. At narrow angles between $4^{\circ }$ and $10^{\circ }$ , even small variations of the twist angle result in large changes in the size of the moiré superlattice. At even smaller angles of $∼1^{\circ }$ , self‐organized reconstruction at an atomic scale into periodic stacking domains may occur.^[13]^

A recent idea is to use the moiré pattern of twisted graphene layers for enantiomer selection or for modifying the properties of confined water molecules.[^14^, ^15^] Furthermore, the adsorption energies of non‐planar methylamine and methanethiol molecules and molecular intercalations on tBLG have been predicted to depend on the twist angle.[^16^, ^17^] For covalent functionalization with diazonium salts, an increased reaction rate has been shown for tBLG compared to an AB‐stacked bilayer.^[18]^ In Ref. [19], a twist‐angle dependent degree of covalent functionalization has been proposed. However, this is based on analysis of the Raman D ‐mode intensity alone, which might also change due to the twist‐angle dependent van‐Hove singularites. Yet, to the best of our knowledge, experimental studies of the dependence of covalent or non‐covalent functionalization on the twist angle in tBLG within the same sample have not been reported.

In this work, we present a novel approach for achieving spatial variations in the degree of non‐covalent functionalization of tBLG by using the moiré lattice as a template for π ‐π stacking. The exfoliated twisted bilayer graphene was non‐covalently functionalized with 1,4,5,8,9,11‐hexaazatriphenylenehexacarbonitrile (HATCN) molecules. HATCN has a planar structure with an aromatic center and six outer cyano groups. Adopting a planar adsorption configuration on graphene, HATCN molecules can only interact with each other through a weak dipolar coupling between their partially negative charged nitrogen atoms and the positively charged carbon atoms in the neighboring cyano groups.[^20^, ^21^, ^22^, ^23^] Therefore, it is expected that the van‐der‐Waals (vdW) and π ‐π interaction with graphene can be stronger for a planar adsorption. The twist angle and degree of non‐covalent functionalization of the tBLG sample are analyzed by Raman spectroscopy. Our results show a correlation of the twist angle and the degree of HATCN functionalization. Thus, the moiré lattice might play a predominant role for the adsorption of the HATCN molecules. Density functional theory (DFT) calculations suggest preferred stacking configurations of HATCN molecules with tBLG, supporting our interpretation of the moiré lattice as template for non‐covalent functionalization.

## Results and Discussion

**Figure 1:**
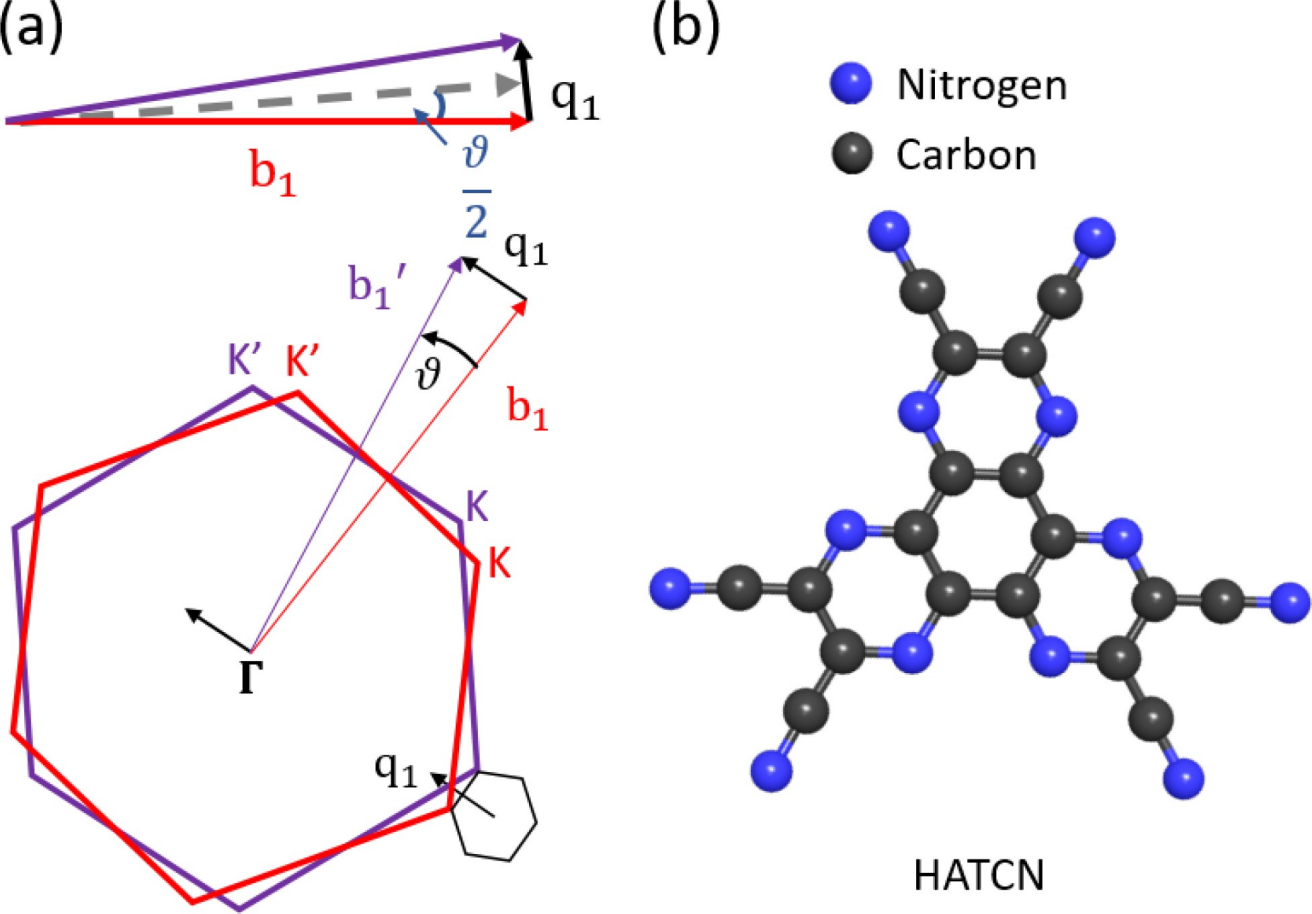
(a) Brillouin zone of twisted bilayer graphene with corresponding mini Brillouin zone and rotational wavevector $q_{1}$ . (b) Molecular structure of 1,4,5,8,9,11‐hexaazatriphenylenehexacarbonitrile (HATCN).

**Figure 2:**
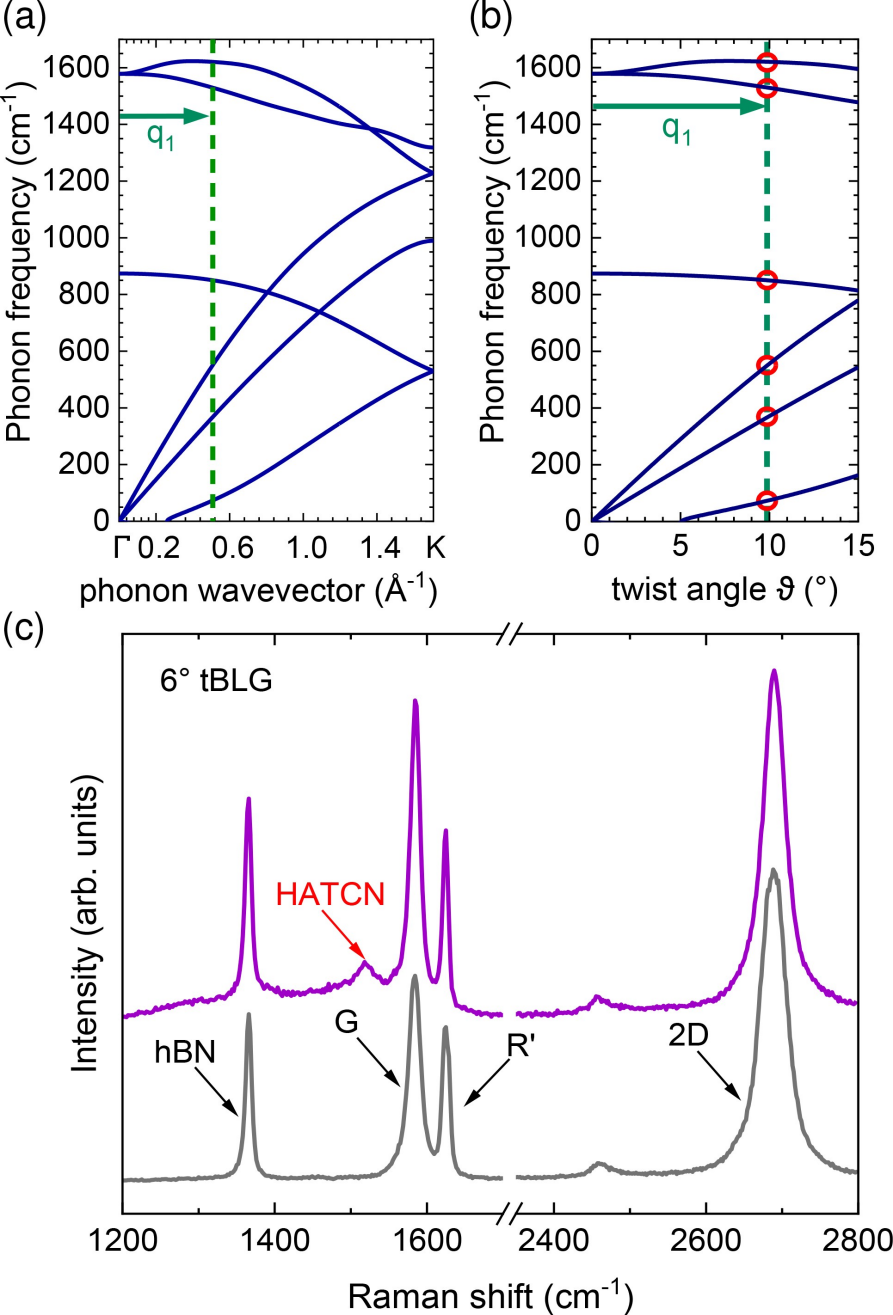
(a) Calculated phonon dispersion of single‐layer graphene. The green arrow indicates the rotational wavevector $q_{1}$ , which represents the difference between the two reciprocal lattice vectors of the two layers in tBLG. (b) Phonon dispersion as a function of twist angle ϑ . Red circles indicate the moiré phonons for a given $q_{1}$ [same as in (a)].(c) Raman spectra (λ=532.17 nm) of a $∼6^{\circ }$ twisted bilayer graphene sample with corresponding 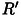 mode, before (grey) and after (purple) HATCN functionalization. The characteristic mode of the HATCN molecule is indicated.

As a first step, we analyze the twist angle of the entire tBLG flake via Raman mapping of the so‐called 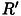 mode. Figure 1 (a) shows the Brillouin zones of rotated bilayer graphene, with the reciprocal lattice vectors of the bottom and top graphene $b_{1}$ and 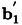 , respectively. Their difference is the reciprocal rotation lattice vector $q_{1}$ , which defines the ‘mini’ Brillouin zone, see Figure 1 (a). For small twist angles ϑ , the direction of $q_{1}$ can be approximated as perpendicular to $b_{1}$ or 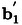 , thus pointing to the ΓK direction of the individual layers’ Brillouin zone. This rotational wavevector $q_{1}$ of the twisted Brillouin zone allows additional Raman scattering away from the Γ point. As an example, the branches of the phonon dispersion intersecting the green line in Figure 2 (a) correspond to Γ ‐point modes in the tBLG and can now be measured in the Raman spectrum. The frequencies of these modes depend on the twist angle; they are often referred to as moiré phonons. The absolute value of the rotational wavevector $q_{1}$ depends on the twist angle [see Figure 1 (a)]:

(1) $$\sin\left(\frac{\vartheta }{2}\right)=\frac{q_{1}/2}{b_{1}}$$

with $b_{1}=\left|b_{1}\right|=\frac{4\pi }{\sqrt{3}a}$ , with a=2.46 Å lattice constant of graphene, resulting in

(2) $$q_{1}\left(\vartheta \right)=\frac{8\pi }{\sqrt{3}a}\sin\left(\frac{\vartheta }{2}\right).$$

Thus, the phonon wavevector along the ΓK direction can be replaced by the twist angle. For larger twist angles $\left(\vartheta >10^{\circ }\right)$ the rotational wavevector $q_{1}$ points more and more towards the ΓM direction, i.e. the conversion of ϑ to a resulting phonon frequency has to be adjusted for certain branches in the phonon dispersion.^[24]^ In this work, tBLG areas with twist angles of around 6° are investigated. Therefore, we use here the graphene phonon dispersion in ΓK direction to derive the ϑ ‐dependent frequencies of the moiré phonons. The 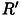 mode ($∼1620\;{\mathrm{cm}}^{-1}$ ), which originates from the longitudinal optical (LO) phonon branch, is the most distinct twist‐induced mode in the Raman spectrum, due to the strong electron‐phonon coupling for the LO branch near the Γ ‐point,^[24]^ see Figure 2 (c). For the determination of the twist angle in our samples, Raman maps of the 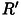 frequency are analyzed and converted to a twist‐angle map, based on the phonon dispersion in Figure 2 (b).

**Figure 3:**
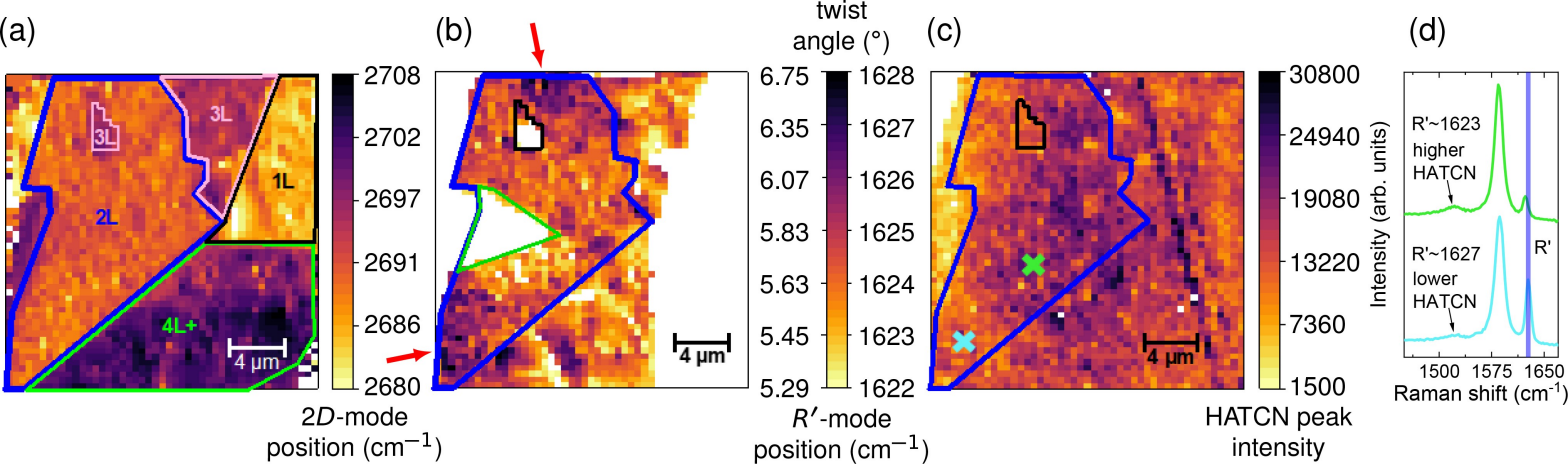
(a) Raman map of the 2*D*‐mode frequency after non‐covalent functionalization. The 2*D* mode was fitted by a single Lorentzian for all graphene layers. Areas with different layer numbers are marked in the map. The area framed by the blue line shows the twisted bilayer (2 L) area. (b) Raman map of the *R′*‐mode frequency and corresponding twist angle after non‐covalent functionalization. The green framed area shows no *R* ^*′*^ mode because the twist angle is smaller than ≲ 5.3°. Red arrows point to areas with higher *R* ^*′*^‐mode frequency and therefore larger twist angle. There is no significant change in the twist angle map before and after chemical functionalization. (c) Raman map showing the intensity of the HATCN peak. The green (high HATCN intensity) and cyan (low HATCN intensity) crosses mark the position of the spectra in Figure 3 (d). (d) exemplary Raman spectra of tBLG with non‐covalent HATCN functionalization, emphasizing the intensity difference of the HATCN peak and the change in frequency of the *R* ^*′*^ mode at different positions on the sample.

Figure 3 (a) shows the Raman map of the 2D ‐mode frequency, indicating the layer number of different areas in our sample. This agrees with the optical microscope image in Supporting Information Figure S1. The area framed by the blue line is the twisted bilayer area. The corresponding 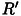 ‐mode position and resulting twist‐angle map is shown in Figure 3 (b), see also Supporting Information Figure S2. We observe twist‐angle variations in our structure between $∼5.3^{\circ }$ and $∼6.7^{\circ }$ . Red arrows point to areas with a twist angle of $∼6.3^{\circ }$ compared to the other parts in the twisted bilayer area with a slightly smaller twist angle of $∼5.5^{\circ }$ . Similar variations of the twist angle within a given tBLG structure have been reported in [25]. The green line indicates an area (white) of the tBLG flake where no 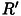 mode was observed. We attribute this to a twist angle below 
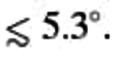
.

To investigate the degree of strain in our tBLG sample, we analyze the peak position of the Raman G mode of graphene, which shows a rather homogeneous distribution across the twisted bilayer area (see Supporting Information Figure S3). From this we exclude that the variations in the 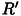 ‐mode frequency [3 (b)] are due to strain.^[26]^ If the shift of the 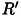 peak of ∼4 cm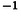 was caused by strain, this would correspond to about ∼0.4 % strain variations in the twisted bilayer area, assuming the same strain‐dependence as for the graphene G mode. Because of the uniform distribution of G ‐mode frequencies across the entire tBLG area, we exclude such strain variations.

Figure 2 (c) shows the Raman spectra from the same tBLG flake before (grey) and after (purple) functionalization with HATCN. After functionalization, there is no increase in the defect‐induced D mode visible, as expected for a non‐covalent functionalization. Instead, a Raman mode belonging to HATCN molecules appears at $∼1520\;{\mathrm{cm}}^{-1}$ (see Supporting Information Figure S4). In order to determine the relative amount of HATCN molecules on the graphene surface, the intensity of the HATCN mode is analyzed in Figure 3 (c). We observe a clear correlation between the HATCN‐mode intensity and the twist angle: Areas with larger twist angle show a lower HATCN‐mode intensity compared to areas with smaller twist angle. Figure 3 (d) shows exemplary spectra from an area with twist angle ∼ 6.3 ° (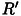 mode at ∼1627 cm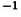 , higher intensity of the HATCN mode) and twist angle ∼ 5.4° (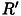 mode at ∼1623 cm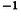 , lower intensity of the HATCN mode). The correlation between the twist angle and the intensity of the HATCN mode can also be observed in other regions of the sample, such as the 3 L and 4 L+ area, which contain the twisted graphene layer. Additionally, in Figure 3 (c) the HATCN‐mode intensity is pronounced along a line across the sample, which can be attributed to a fold in the hBN layer, see the optical microscope image in Supporting Information Figure S1. Here, we observe also a high intensity of the HATCN signal. This might be attributed to a weaker attachment of the graphene to the hBN next to the fold. We recorded Raman maps of the tBLG sample before and after non‐covalent functionalization. The 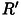 ‐mode position remained unaffected, indicating that no notable changes in the twist‐angle distribution were introduced by the functionalization procedure.

**Figure 4:**
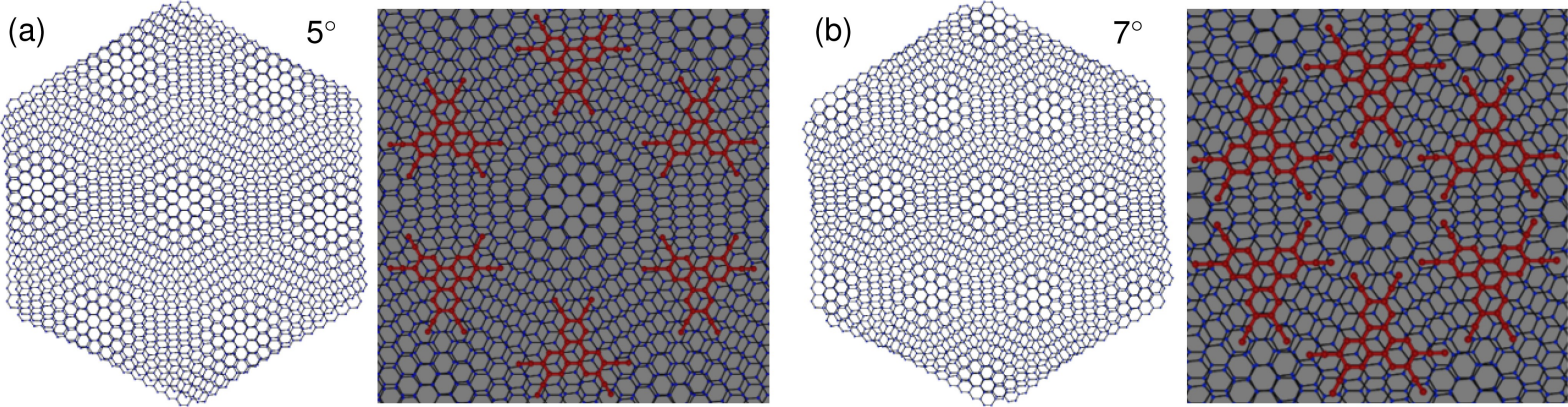
(a) and (b); moiré lattice of 5° and 7° twisted bilayer graphene, respectively. Schematic views of possible arrangements of HATCN molecules (red) on AB‐stacked regions, showing different densities of the HATCN molecules for the two twist angles. In the schematic view of the 7° moiré lattice, the nitrogen atoms of the CN groups are too close to each other. As a result, the HATCN molecules cannot attach to each AB‐stacked region. For additional arrangements see Supporting Information Figure S9.

The correlation between twist angle and the HATCN‐mode intensity points to a twist‐angle dependent arrangement of the HATCN molecules on the tBLG. We expect a low coverage of HATCN molecules, leading to a ‘planar’ monolayer adsorption on the graphene surface (also due to hBN, see Supporting Information Figure S5), indicating a suppressed molecule‐molecule interaction.^[21]^ In Figure 4 we show the geometries of the moiré lattices for twist angles of $5^{\circ }$ and $7^{\circ }$ , the upper and lower boundaries of twist angles in our sample.

For the same area, the number of AA‐stacked and AB‐stacked regions at $7^{\circ }$ (right) is higher than at $5^{\circ }$ twist angle (left). The moiré superlattice for $5^{\circ }$ ($7^{\circ }$ ) has a periodicity of 2.82 nm (2.02 nm). The size of the moiré unit cell for $7^{\circ }$ coincides in fact with the unit cell of the self‐assembly of HATCN on Ag(111).^[20]^ Since the functionalization mechanism is predominately based on π*–*π stacking, the molecules will preferably choose either locally AA‐stacked or AB‐stacked regions within the moiré lattice, thus following its size and the electronic charge density difference of tBLG, which varies spatially.^[14]^ Initially, the fact that the $7^{\circ }$ moiré lattice and the self‐assembly lattice constants match, could suggest the formation of this self‐assembly on tBLG. However, this would only be possible if the HATCN molecules occupy either AA‐stacked sites or every second AB‐stacked site (due to spatial limitations). Second, this would imply that the intermolecular interaction is considerably larger than the molecule‐graphene interaction. An investigation of the total energy (SI Figure S6) confirms that the HATCN molecules seem to avoid AA‐stacked regions and tend to reside on AB‐stacked bilayer graphene regions, see DFT calculations below. This is consistent with the measurements at a twist angle of $5^{\circ }$ , which show a higher degree of functionalization, since the distance between two AB‐stacked regions is large enough to accommodate HATCN molecules on each of the AB‐stacked sites, see Figure 3.

Our DFT calculations (see Supporting Information for computational details) of the charge density redistribution of HATCN on bilayer graphene (see Supporting Information Figures S7 and S8) show the highest interaction strength of HATCN molecules which are placed on AB‐stacked bilayer graphene to form an ABA configuration. Additionally, the energy difference per HATCN between the optimal ABA configuration and a less suitable ABB arrangement is on the order of 0.1 eV. This makes a significant change of the molecular arrangement, including a transition to the self‐assembly configuration, unlikely.

Figure 4 schematically shows a possible configuration of HATCN molecules with an AB‐stacking of the HATCN centering on AB‐stacked tBGL domains. The distance between the nitrogen atoms at the outer wings of the molecules remains in the order of several Å for the $5^{\circ }$ twist angle configuration. At $7^{\circ }$ , the distance between the nitrogen atoms of neighboring HATCN molecules decreases to around 1–2 Å. As a result the HATCN molecules cannot attach to every AB‐stacked region on the $7^{\circ }$ moiré lattice since the partially negatively charged nitrogen atoms of the CN groups are too close to each other, see Supporting Information Figure S9.

The fact that the experimental HATCN‐mode intensity is higher at $5^{\circ }$ than at $7^{\circ }$ , indicates that the surface‐molecule interaction dominates over the molecule‐molecule interaction, which also aligns with our DFT calculations. The hypothesis of preferential attachment of HATCN molecules on AB‐stacked regions in tBLG is in agreement with the HATCN molecule distribution shown in Figure 3 (c). This clearly indicates that the moiré lattice of tBLG can affect the non‐covalent interaction of molecules on graphene.

To further test our hypothesis, we performed DFT calculations of 6×6 bilayer graphene supercells in which a single HATCN molecule was placed (see Supporting Information for computational details). The starting arrangement of bilayer graphene was chosen to be either AA‐ or AB‐stacked. The HATCN molecule was placed on top of the bilayer graphene such that the center of the molecule aligns with the upper graphene layer in either AA or AB configuration, resulting in overall AAA‐, AAB‐, ABB‐, and ABA‐stacking configurations, see Supporting Information Figure S10.

After relaxation of the structures, an investigation of the total energy reveals that the energetically most favorable arrangement exhibits an ABA‐stacking order, which arises from the two configurations in which the HATCN molecule is placed in an AB arrangement on the top layer of both AA‐ and AB‐stacked bilayer graphene. Note that throughout the relaxation process of the AA‐stacked bilayer graphene with an HATCN molecule placed in an AB configuration, the interaction between the molecule and the graphene is strong enough to induce a transition in the bilayer graphene from an AA‐ to an AB‐like configuration (see Supporting Information Figure S10). This results in a total energy comparable to that of the ABA‐stacking arrangement, which exhibits the lowest total energy.

The highest total energy is found for the AAA‐stacking configuration (see Supporting Information Figure S6). In contrast to the supercell described above where the HATCN molecule is in AB configuration on top of AA‐stacked graphene, no major structural changes occur during the relaxation process. This hints at a lower interaction strength between the molecule and the AA‐stacked graphene.

In addition to the 6×6 supercells, we also constructed a 5×5 and a 7×7 supercell, each containing one HATCN molecule (all in ABA configuration). The relaxation of these structures gives further insight into the closest possible arrangement of neighboring HATCN molecules. The 5×5 cell seems to be insufficient for the accommodation of an HATCN molecule, which leads to a partial detachment of the molecule (see Supporting Information Figure S11). This is furthermore reflected by the fact that the energy per HATCN is even higher than the energy per HATCN for the unfortunate AAA configuration, see Supporting Information Figure S7. The energy per HATCN of the 7×7 structure matches the energy of the corresponding 6×6 configuration, hinting at the fact that larger distances between the molecules do not result in significantly less favorable arrangements.

The interaction between HATCN and tBLG is confirmed by calculating the redistribution of the charge density of individual HATCN molecules on bilayer graphene. The charge densities of isolated bilayer graphene and isolated HATCN molecules were subtracted from the charge density of the entire structure. Much more additional charge density between graphene and HATCN is found for the ABA‐configuration compared to other arrangements (see Supporting Information Figure S7).

Our DFT calculations of freestanding HATCN confirm the self‐assembly, however with a unit cell of 1.88 nm (see Supporting Information Figure S12). The distances between neighboring CN‐groups remain larger than 3.5 Å. The atoms of the HATCN molecules were artificially kept within the xy ‐plane, since a relaxation would otherwise not have been possible. This furthermore illustrates the high importance of the interaction of HATCN with the substrate.

Our DFT results clearly indicate stronger interactions for an AB‐alignment of the HATCN molecule on top of graphene, which suggests that this is the more favorable arrangement compared to AA‐stacking. Additionally, because of the significantly lower total energy of the ABA configurations, this seems to be the most favorable one. These results hint at the preferred formation of an ABA arrangement of tBLG and HATCN molecules, in agreement with the experimental data.

To provide further support for our experimental observations, we conducted Kelvin probe force microscopy (KPFM) measurements. The KPFM measurements also indicate a correlation between the surface potential of the sample and the HATCN intensity, comparable to the results of our Raman measurements. For further details, see Supporting Information Figures S13 and S14.

**Figure 5:**
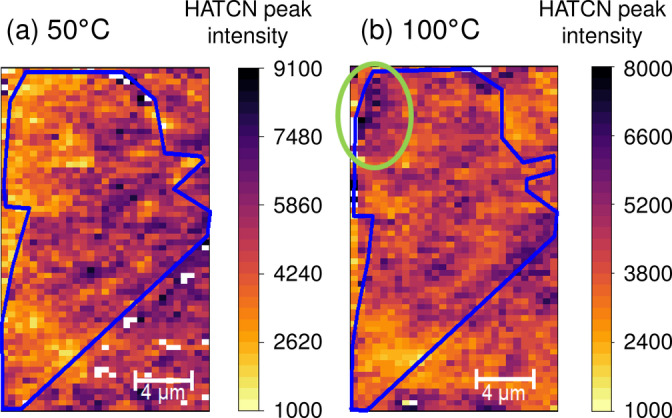
Comparsion of the HATCN peak intensity distribution at 50 °C (left) and 100 °C (right) of the same tBLG sample shown in Figure 3. A clear increase of the HATCN peak intensity is observed in the upper left part of the twisted bilayer area (green circle) at 100 °C.

To obtain more insight into the stability of the HATCN molecule adsorption, we performed temperature‐dependent Raman measurements at elevated temperatures. The Raman maps of the twist angle distribution (see Supporting Information Figures S15–S19) show no significant difference at temperatures up to 200 °C compared to room temperature [3 (b)]. Figure 5 shows a comparison of the HATCN‐mode intensity maps at 50 °C (a) and 100 °C (b). At higher temperatures, the HATCN molecules become more mobile and are able to move on the graphene surface; in Figure 5 (b), the intensity of the HATCN signal changes locally. For example, the degree of HATCN functionalization increases locally in the area marked by the green circle. In the other parts, the distribution is still similar to the 50 °C and the room‐temperature Raman map. The area showing now higher HATCN signal, also exhibits a slightly smaller twist angle, leading to larger distances between the HATCN molecules and therefore to a preferred attachment. Thus, the HATCN molecules seem to still arrange themselves through the influence of the underlying moiré lattice and the correlation between twist angle; the degree of the HATCN functionalization is still visible in the Raman maps at 100 °C. At higher temperatures, a homogeneous distribution of HATCN molecules in the tBLG region is observed (SI Figure S19). Further temperature‐dependent Raman maps, as well as maps showing the twist angle and the HATCN‐mode intensity after a second HATCN functionalization are shown in the SI.

The question might arise whether the HATCN molecules are intercalated between the graphene layers or between the bottom hBN and tBLG. We therefore prepared functionalized graphene with an additional top hBN layer (after functionalization). Temperature‐dependent Raman measurements (SI Figure S21) show that the HATCN mode is still present at 300 °C, in contrast to our previous measurements without the top hBN layer. This indicates that the HATCN molecules do not intercalate but are indeed on top of the tBLG structure.

In order to rule out an accidential inhomogeneity of the HATCN‐molecule distribution on our tBLG samples, we performed the same non‐covalent functionalization procedure on single‐layer and natural bilayer graphene. These measurements show a homogeneous intensity distribution of the HATCN Raman signal, indicating a homogeneous coverage of HATCN molecules (see Supporting Information Figure S22). For bilayer graphene, we observe an additional influence of the underlying substrate: The total intensity of the HATCN mode is lower for graphene on hBN than on ${\mathrm{SiO}}_{2}$ (see Supporting Information Figure S5). This is due to the chemical inertness of hBN and is in line with our previous investigations[^5^, ^27^] on covalently functionalized graphene, showing that the reactivity of the underlying substrate plays an important role also for the degree of the non‐covalent functionalization. Investigations of the exact arrangement of the HATCN molecules on the moiré lattice by atomic‐scale techniques, such as scanning tunnelling microscopy (STM) or tip‐enhanced Raman spectroscopy (TERS) would be of interest. However, ambient STM or TERS measurements would require the fabrication of structures with very small twist angles ($\leq 0.5^{\circ }$ ) and thus large moiré superlattice periodicity, in which AB‐ and AA‐stacked areas can be clearly distinguished from each other.^[28]^ For such small twist angles, on the other hand, the R or 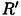 Raman modes would not be detectable, thereby impeding the determination of twist‐angle variations by Raman spectroscopy.

## Conclusions

In summary, we present a novel approach to achieve spatial variations in the degree of non‐covalent functionalization of bilayer graphene by using the moiré lattice of tBLG as a template. Our results show a clear correlation between the twist angle of tBLG and the degree of functionalization with HATCN molecules. Our assumption of a preferred attachment of the HATCN molecules in AB‐stacked regions, resulting in a local ABA‐stacking with the twisted graphene layers, is supported by DFT calculations. Moiré structures of other 2D materials may be used as well for nanoscale patterning of non‐covalent functionalization. The ability to precisely control the spatial attachment of functional groups by tuning the twist angle in twisted bilayer materials provides new possibilities in the field of high‐precision nanoscale materials engineering.

## Supporting Information

The authors have cited additional references within the Supporting Information.[^29^, ^30^, ^31^, ^32^, ^33^, ^34^, ^35^, ^36^, ^37^, ^38^, ^39^, ^40^, ^41^]

## Conflict of Interests

The authors declare no conflict of interest.

1

## Data Availability

The data that support the findings of this study are available on request from the corresponding author. The data are not publicly available due to privacy or ethical restrictions.
